# Variation in the quality of opioid use disorder treatment in the Medicaid population in 2019

**DOI:** 10.1371/journal.pone.0341739

**Published:** 2026-03-04

**Authors:** Ryan M. Andrews, Vishwali Mhasawade, Selena Maity, Anton Hung, Richard Liu, Rachael K. Ross, Hillary Samples, Kara E. Rudolph

**Affiliations:** 1 Department of Epidemiology, Columbia University Mailman School of Public Health, New York, New York, United States of America,; 2 NYU Langone Health, New York, New York, United States of America; 3 Department of Health Policy Management, Columbia University Mailman School of Public Health, New York, New York, United States of America; 4 Division of Biostatistics, Department of Population Health, NYU Grossman School of Medicine, New York, New York, United States of America; 5 Center for Pharmacoepidemiology and Treatment Science, Rutgers Institute for Health, Health Care Policy and Aging Research, New Brunswick, New Jersey, United States of America; Virginia Commonwealth University School of Medicine, UNITED STATES OF AMERICA

## Abstract

**Background:**

This study aimed to describe state- and urbanicity-stratified differences in three opioid use disorder (OUD) treatment metrics among Medicaid beneficiaries in 25 states that implemented Medicaid expansion under the Affordable Care Act by the end of 2014.

**Methods:**

Using data from 2019, we identified Medicaid beneficiaries with OUD based on ICD-10 diagnosis codes. We then calculated the percentage of beneficiaries who met criteria for three metrics measuring OUD treatment quality, both overall and stratified by state and urbanicity type. The OUD treatment quality metrics considered were: (1) initiation of medication for opioid use disorder (MOUD) treatment, (2) engagement with OUD services, and (3) retention on MOUD treatment.

**Results:**

Across states, we found that a median of 26.2% of beneficiaries initiated MOUD within 14 days of their OUD diagnosis date in the claims data. A median of 15.8% of beneficiaries engaged with OUD treatment services by initiating MOUD treatment within 14 days of their OUD diagnosis and receiving at least 2 distinct OUD-related services within 30 days of their MOUD initiation date. Among initiators, a median of 30.8% were retained on MOUD treatment for a minimum of 180 days. However, there was considerable heterogeneity in these three metrics across states; New Hampshire and West Virginia were found to have the highest overall performance out of the states considered. With respect to urbanicity, we found that rural and suburban areas had higher percentages of beneficiaries who met our three treatment quality metrics compared to urban areas.

**Conclusions:**

We found notable geographic differences in opioid use disorder treatment quality in the U.S. Medicaid population.

## Introduction

Opioid use disorder (OUD) is a major risk factor for several adverse outcomes, including fatal overdoses, all-cause mortality, and engagement with the criminal justice system [1]. Medications for OUD (MOUD) effectively reduce risk of relapse, overdose, and death. However, research shows that initiation of and retention on MOUD is low, likely due to a number of substantial structural barriers [2–4]. In response, some burdensome requirements of opioid treatment programs that were relaxed during the COVID-19 pandemic recently became permanent, including telehealth, take-home medication supply, and prescribing flexibilities [5,6]. Nevertheless, access to MOUD treatment has been found to vary across the United States. For example, although studies suggest that federal policy reforms have increased the overall reach of MOUD (particularly buprenorphine) [7,8], other studies have observed state-by-state differences in MOUD prescribing patterns and access, even after these policies went into effect [4,9–16]. Therefore, ongoing research is needed to improve understanding of state-level differences that can facilitate development of tailored policies, programs, or health system infrastructure to address gaps in MOUD treatment.

In addition, studies have shown that despite rural areas experiencing significantly more drug overdose deaths and opioid-related harms compared to urban areas, OUD patients living in rural areas face more barriers to receiving MOUD treatment (e.g., provider shortages and fewer MOUD-dispensing healthcare facilities compared to patients living in more urban areas), even with ongoing efforts to expand OUD-related services (including MOUD) in these areas [17–22]. Taken together, this suggests that aggregate summaries of MOUD treatment patterns may overlook important geographic disparities that could be targeted with future interventions.

Relatedly, Medicaid is the largest payer for OUD services in the United States, covering 40–50% of all non-elderly OUD patients [23,24]. Because Medicaid operates as a federal-state partnership, there are documented differences in access and delivery of MOUD across different states’ Medicaid populations [13,14,25]; there are similar reports of differences in MOUD access and delivery for Medicaid patients residing in urban versus rural areas [15,26]. Therefore, capturing geographic-related heterogeneity in MOUD treatment within the Medicaid system may be policy-relevant.

In this study, we describe state- and urbanicity-stratified differences in three MOUD treatment metrics among Medicaid beneficiaries in 25 states that implemented Medicaid expansion under the Affordable Care Act by the end of 2014. Specifically, we considered three quality metrics related to MOUD treatment from the National Quality Forum that are measures of 1) initiation of MOUD, 2) engagement in care, and 3) retention in MOUD [27,28]. These aspects of OUD treatment are three out of the four components of the treatment cascade within the OUD Cascade of Care Model, which is a public health framework for identifying targets at the local and national level to improve the prevention and treatment of OUD and track the progress of these efforts [28,29].

## Methods

Our study included de-identified data from Medicaid T-MSIS Analytic Files (TAF): Demographics, Other Services for 2019. These data include adult, non-dual eligible Medicaid beneficiaries aged 19−64, and we restricted our sample to those diagnosed with OUD (based on ICD-10 diagnosis codes) in 25 states that implemented the Affordable Care Act (ACA) by 2014: AR, AZ, CA, CT, CO, DE, HI, IA, IL, KY, MA, MD, MI, MN, ND, NH, NJ, NM, NY, OH, OR, RI, VT, WA, WV; MD was excluded due to unreliable diagnosis code data, as described in the Medicaid Data Quality Atlas) [30]. We included these states because they covered nearly all non-elderly, low income (< 138% of the Federal Poverty Limit) adults, and the group that was newly eligible for Medicaid under the ACA represents a high-risk group for OUD (e.g., younger, male, and low-wage workers) [31,32]. We classified beneficiaries’ residential county as urban, suburban, or rural using Rural-Urban Continuum Codes (RUCC). Patients missing a RUCC classification were excluded from analyses stratified by urbanicity. All analyses were conducted between March 1, 2025 and July 31, 2025.

To measure OUD treatment quality, we used three metrics: (1) the percentage of patients with an OUD diagnosis who were prescribed buprenorphine, extended-release injectable naltrexone, or methadone within 14 days of their OUD diagnosis (hereafter, “MOUD initiation”); (2) the percentage of patients with an OUD diagnosis who initiated MOUD within 14 days of their OUD diagnosis *and* received at least 2 services within 30 days of their MOUD initiation (hereafter, “engagement with OUD services”); (3) the percentage of patients initiating MOUD treatment who received at least 180 days of continuous MOUD therapy, where continuous therapy was defined as not having a gap in MOUD of more than 7 days (hereafter, “retention in MOUD treatment”) [27,33,34]. These treatment measures comprise key components of the OUD cascade of care, which tracks progress through an evidence-based treatment episode [28,29].

Each of these percentages were defined as the number of Medicaid beneficiaries who met specific OUD treatment criteria divided by the total number of beneficiaries who were eligible to meet these criteria; Table 1 provides details on the numerator and denominator for each treatment quality metric. For each metric, we calculated both pooled (using all 25 states) and state- and urbanicity-stratified percentages.

**Table 1:** Numerator and denominator definitions for the 3 opioid use disorder treatment quality metrics of interest among 2019 Medicaid beneficiaries.

Quality metric	Eligibility criteria for numerator	Eligibility criteria for denominator
Initiation of MOUD treatment	Patients who were prescribed buprenorphine, extended-release injectable naltrexone, or methadone (identified by a variety of procedure and drug codes) within 14 days of their OUD diagnosis	Patients who received an OUD diagnosis who were continuously enrolled in Medicaid for at least 14 days after their OUD diagnosis
Engagement with OUD services	Patients who initiated MOUD treatment within 14 days of their OUD diagnosis and received at least 2 services (distinct outpatient, inpatient, or emergency department claims with an OUD diagnosis code) within 30 days of their MOUD initiation	Patients who received an OUD diagnosis who were continuously enrolled in Medicaid for at least 44 days after their OUD diagnosis
Retention in MOUD treatment	Patients who received at least 180 days of continuous MOUD therapy, where continuous therapy was defined as not having a gap in MOUD of more than 7 days	Patients who were diagnosed with OUD, initiated MOUD, and who were continuously enrolled in Medicaid for at least 180 days after initiating MOUD

OUD = opioid use disorder; MOUD = medication for opioid use disorder.

This study was approved by the Columbia University Institutional Review Board. All analytic code to replicate the analyses is available at: https://github.com/CI-NYC/OUD-tx-state-year-variability, along with all diagnostic codes used to identify OUD-related Medicaid claims.

### Results

**Fig 1:**
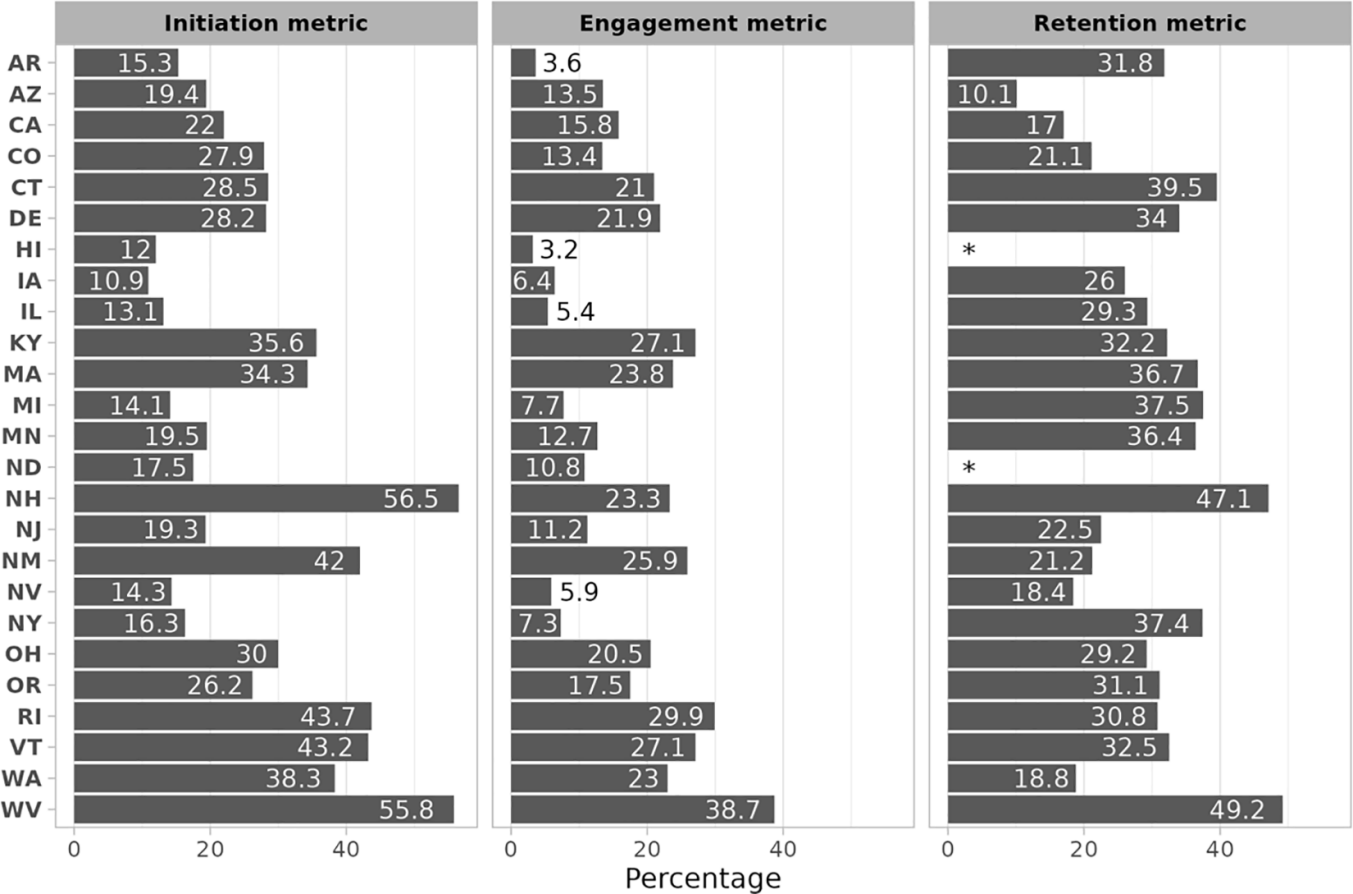
Distribution of OUD treatment metrics in 2019 among Medicaid beneficiaries residing in 25 US states that expanded Medicaid under the Affordable Care Act. OUD = opioid use disorder. * Indicates that data were excluded due to small cell sizes.

**Fig 2:**
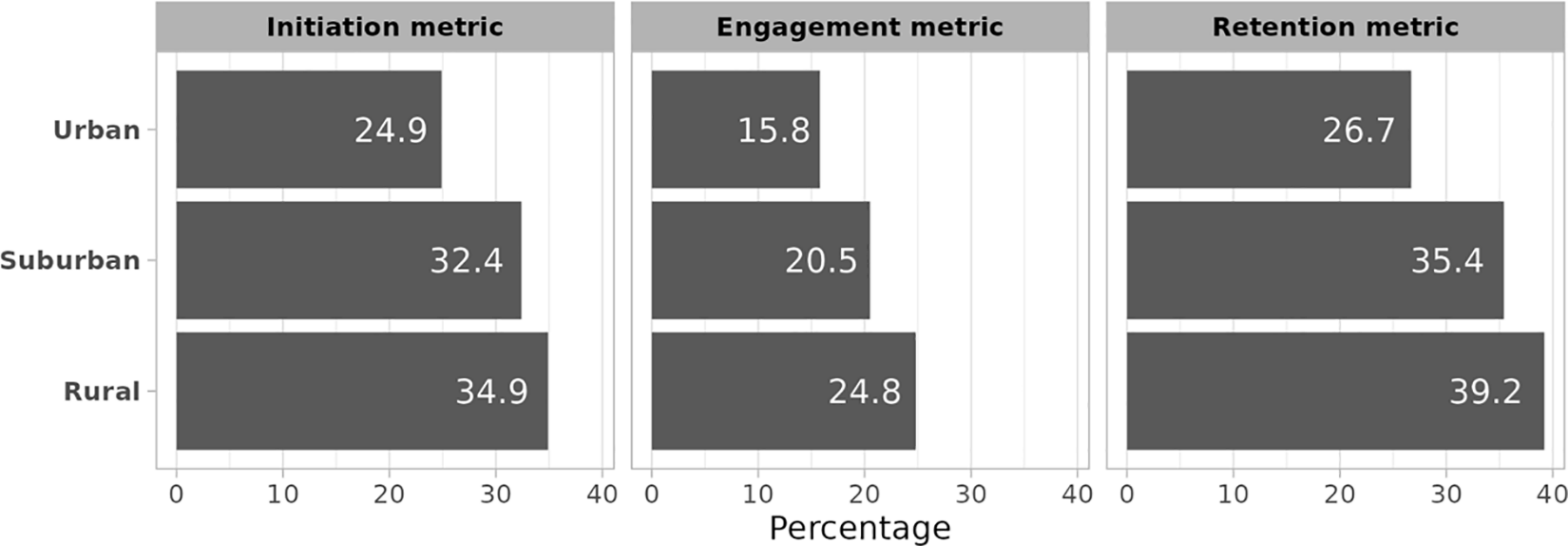
Distribution of OUD treatment metrics in 2019 by urbanicity among Medicaid beneficiaries residing in 25 US states that expanded Medicaid under the Affordable Care Act. OUD = opioid use disorder.

The median percentages and the interquartile range (IQR) of each treatment quality metric across states are presented in Table 2. The distributions of the percentage meeting each quality metric across states and urbanicity groups are represented in Figs 1 and 2, respectively. Additional details, including state- and urbanicity-specific cell counts, are given in S1 and S2 Tables, respectively.

**Table 2:** Descriptive statistics for the 3 opioid use disorder treatment quality metrics of interest among 2019 Medicaid beneficiaries, overall and across states.

	Percentage of patients	
Quality Metric	Overall (25 states pooled)	Median (IQR) across states
**Initiated MOUD within 14 days of OUD diagnosis**	26.4%	26.2% (16.3, 35.6)
**Engaged in care within 44 days of OUD diagnosis**	16.9%	15.8% (7.7, 23.3)
**Retained in MOUD treatment for at least 180 days, among those initiating MOUD treatment**	29.2%	30.8% (22.0, 36.4)

OUD = opioid use disorder; MOUD = medication for opioid use disorder; IQR = interquartile range.

We estimated that a median of 26.2% of Medicaid beneficiaries in our sample with OUD (N = 155,135) initiated MOUD treatment within 14 days of their diagnosis, with an IQR of 16.3–35.6% across all 25 states (Table 1). Iowa was found to have the lowest percentage of MOUD initiators (10.9%), while West Virginia (55.8%) and New Hampshire (56.5%) had the highest (Fig 1). When we stratified by urbanicity, we found that 5,221 (3.4%) beneficiaries had missing RUCC codes and were therefore excluded. Among the remaining 149,914, approximately one third of those living in rural and suburban areas initiated MOUD treatment within 14 days of a diagnosis (34.9% and 32.4%, respectively); and approximately one quarter (24.9%) of those living in an urban area did (Fig 2).

We found that a median of 15.8% (out of 142,746 beneficiaries eligible) engaged with OUD services within 44 days of their diagnosis, with an IQR of 7.7–23.3% across 25 states (Table 2). West Virginia had the highest level of engagement with services (38.7%), while Hawaii had the lowest (3.2%). When looking at engagement by urbanicity, we found that 4,783 (3.4%) beneficiaries had a missing RUCC code and were excluded. Among the remaining 137,963 beneficiaries, those in rural areas had the highest engagement with OUD services (24.8%) compared to those in suburban (20.5%) and urban (15.8%) areas (Fig 2).

When considering retention in MOUD treatment, we excluded data from two states – Hawaii and North Dakota—because of small cell sizes. Among the remaining 23 states, we found that a median of 30.8% of those who initiated MOUD (N = 27,959) were retained on MOUD for at least 180 days, with an IQR of 22.0–36.4% across the 22 states (Table 1). Arizona was found to have the lowest retention percentage (10.1%), whereas New Hampshire (47.1%) and West Virginia (49.1%) were found to have the highest percentages (Fig 1). Stratifying by urbanicity, we found that 682 (2.4%) were missing a RUCC code and were excluded. Among the remaining 27,277 beneficiaries, the percentage of those retained in MOUD treatment was 39.2% for rural areas, 35.4% for suburban areas, and 26.7% for urban areas (Fig 2).

## Discussion

This descriptive study aimed to estimate MOUD treatment quality metrics among adult Medicaid patients in 2019 across 25 states that expanded Medicaid eligibility under the ACA by 2014. These metrics captured three out of the four components of the treatment cascade within the OUD Cascade of Care model. We found that approximately one-fifth of beneficiaries engaged with MOUD treatment services per the definition (initiated MOUD treatment within 44 days of their OUD diagnosis and received at least 2 services within 30 days of their MOUD initiation). Percentages of initiation and retention in MOUD treatment were higher, with approximately a quarter of beneficiaries meeting criteria across all 25 states. However, there was noticeable variability across states in the percentage of beneficiaries who met the metrics’ criteria. At the same time, there was moderate to high correlation between the relative ranking of each state according to these percentages, indicating that states that perform well according to one MOUD treatment metric tend to perform well on others. When looking across urbanicity subgroups, we found that rural areas had the highest percentage of beneficiaries meeting MOUD treatment criteria (followed closely by suburban areas), while urban areas had the lowest.

Our overall results are largely consistent with values reported by other studies. One study examining commercially insured patients in 2014 used an Initiation metric identical to ours and reported a slightly higher percentage of 40.9% in its OUD-specific subgroup [35]. This study also reported an engagement metric of 21.6%, slightly higher than in our study. Among Medicaid enrollees across 11 states from 2014 to 2018, another study reported the percentage of MOUD patients being retained on MOUD treatment for a continuous 180 days as 47.8% −57.1%, which was considerably higher than we found [15]. Given the heterogeneity between states that we observed, it is possible that this difference is at least partially driven by the different states considered (comparing [15] with our study, we examined only 5 of the same states – Delaware, Kentucky, Michigan, Ohio, and West Virginia).

We observed state-to-state variability in all three metrics. Similar variability was reported across 11 states by Brown et al., who propose that this variability may be driven by demographic differences [15]. Another explanation for this variability could be state-specific differences in availability of providers and treatment programs. Relatedly, the Drug Addiction Treatment Act of 2000 required that healthcare providers obtain a waiver to prescribe buprenorphine (called the X waiver). This waiver was required for physicians and some advanced practitioners who received training in addiction medicine, and once the waiver was obtained, the number of patients who could be prescribed buprenorphine was capped at 30 for the first year (and up to 275 thereafter) [36]. These administrative hurdles may have differentially affected MOUD across states in 2019, due to, for example, primary care physician shortages. Another explanation for the observed state-by-state heterogeneity in OUD treatment quality could be differences in state-level initiatives targeting OUD. It is notable that West Virigina and New Hampshire consistently were ranked highly for MOUD initiation, engagement, and retention, given that these states also rank among the hardest hit by the opioid epidemic. For example, it is estimated that West Virginia had the highest opioid-related overdose death rate in the United States in 2017 (49.6 per 100,000 residents) and New Hampshire had the third highest rate during this same period (34.0 per 100,000 residents) [37]. Increased awareness of overdose deaths may have brought OUD and MOUD treatment greater attention, which may have resulted in a greater percentage of those with OUD seeking treatment in these states. Relatedly, both West Virginia and New Hampshire introduced policies to combat OUD and expand access to MOUD. In West Virginia, the Comprehensive Opioid Addiction Treatment (COAT) program started in 2005 to provide evidence-based care to those diagnosed with an OUD by combining MOUD management consultations with group-based therapy to treat OUD. Studies have shown that the COAT program has high patient retention, and by treating patients in groups (versus individually), COAT addresses some of the roadblocks to treatment in a predominantly rural state (e.g., provider shortages) [38]. Relatedly, in 2018, West Virgina’s Medicaid began covering methadone for the first time, resulting in a large number of patients receiving OUD diagnoses and MOUD treatment [39]. In New Hampshire, a hub-and-spoke system called “The Doorway” was introduced in 2019 with funding from SAMHSA to improve access to treatment, recovery, and harm-reduction services for individuals with an OUD or other substance use disorder [40]. Overall, The Doorway improved many aspects of OUD treatment in New Hampshire, including expanded access to MOUD. Of relevance to our study, the majority of patients served by The Doorway were Medicaid patients [40]. However, it is important to note that although it is likely that these policies enacted around the time of our data collection helped patients with an OUD in West Virginia and New Hampshire, we have insufficient evidence to claim that these programs are the reason for them being top performers in our study relative to other states.

Our urbanicity-stratified results diverged from previous literature, which had found that rural areas fell behind urban areas in terms of MOUD treatment. For example, a recent systematic review of 18 studies found that those in rural areas had to travel farther to access MOUD and to see OUD treatment providers, which was associated with lower MOUD treatment for individuals with an OUD living in rural areas [18]. Another study found that although rural and urban hospitals had similar rates of OUD screening, those in rural areas were less likely to offer MOUD and other addiction services [17]. In addition, a large study using data from the 2021 National Survey on Drug Use and Health (NSDUH) found that OUD patients residing in non-metropolitan areas had 69% lower odds of receiving MOUD compared to those in metropolitan areas [41]. There could be several potential explanations for why our results diverged from prior studies showing differences in MOUD treatment by urbanicity. One possibility is that primarily rural states with state-wide, OUD-focused programming (e.g., West Virginia and New Hampshire, as mentioned above) led us to find that rural and suburban areas perform better than urban areas in terms of OUD treatment quality. Another possibility is related to our focus on the Medicaid population; rural buprenorphine (a type of MOUD) prescribers are more likely than urban prescribers to accept Medicaid insurance [42]. A third possibility is that, given the barriers to healthcare in rural areas described above, patients living in rural areas with OUD may be less likely to receive an OUD diagnosis than patients living in urban areas with OUD. This urban-rural discrepancy could be especially pronounced for Medicaid-insured patients given that barriers to accessing care would be steeper in the context of socioeconomic challenges. If true, then we would expect the patient populations comprising the denominators of our metrics to differ between urban and rural areas. For example, Medicaid patients in rural areas who have an OUD diagnosis in the claims data may be more treatment-motivated, on average, than otherwise clinically similar Medicaid patients in urban areas who have an OUD diagnosis in the claims data. Thus, our results more accurately describe MOUD treatment quality among rural OUD patients who have been identified and diagnosed with OUD. These conjectures and limitations should be explored in additional studies, including model-based studies that aim to provide empirical evidence for the drivers of MOUD treatment quality by urbanicity.

A strength of our study is that we were able to include Medicaid beneficiaries from 25 states in the US, which allowed us to explore both state and geographic trends in MOUD treatment. Relatedly, because the Medicaid expansion population comprises a high-risk group for OUD (e.g., low-income, childless adults), studying OUD treatment quality within this sample allowed us to capture trends that likely have high relevance for MOUD treatment. Another strength of our study is the reporting of multiple treatment metrics from the OUD Cascade of Care model. Although prior work has used this framework to study OUD and MOUD treatment in electronic health records [43], to our knowledge our study is the first to report on these metrics within a purely Medicaid patient sample.

However, there are several important limitations to our work. First, our results may not generalize to different populations of OUD patients outside of the 25 state Medicaid samples that we used. Second, our results are based on cross-sectional data from 2019, which prevented us from identifying new OUD diagnoses and examining trends in MOUD treatment quality over time. Relatedly, Medicaid accessibility, funding, and policy are constantly evolving, which may limit our ability to interpret 2019 findings as being representative of OUD treatment quality for Medicaid beneficiaries in the present day. This is especially true given key policy changes that took effect after 2019: 1) the Consolidated Appropriations Act, enacted in 2023, eliminated the need for providers to apply for the “X waiver” to prescribe buprenorphine for the treatment of OUD; 2) allowance of MOUD to be prescribed via telehealth, initiated during the Covid pandemic in 2020 and subsequently made permanent; and 3) allowance of take-home methadone doses (up to 28 days for stable patients), also initiated in 2020 and made permanent [5,6]. However, an additional fourth policy, implemented in 2025, increases the relevance of 2019 data, because it rolled back the Covid emergency exemption that paused all disenrollment from the Medicaid due to administrative reasons [44]. Thirty percent of those who lose Medicaid coverage do so for administrative reasons, termed “churn” [44,45] Consequently, generalizability of Medicaid data after 2019 would be compromised for this reason. Finally, our study was by design descriptive, and the percentages we report were not adjusted for any variables; as such, our results should not be interpreted as describing any causal relationships involving MOUD treatment quality, differences in state Medicaid policies, and/or differences in MOUD treatment access across urban, suburban, and rural areas.

## Supporting information

S1 Table Raw counts (numerators and denominators) for the three MOUD treatment quality metrics reported in the main paper by state. Due to small cell counts, some data are not reported (indicated by an NA). OUD = opioid use disorder; MOUD = medication for opioid use disorder.
(PDF)

S2 Table Raw counts (numerators and denominators) for the three MOUD treatment quality metrics reported in the main paper by urbanicity type. OUD = opioid use disorder; MOUD = medication for opioid use disorder.
(PDF)
