# Supplementary material for: Variation in the quality of opioid use disorder treatment in the Medicaid population in 2019
Source: PLoS One. 2026 Mar 4;21(3):e0341739. doi: 10.1371/journal.pone.0341739 (PMC12959680; doi:10.1371/journal.pone.0341739)
Supplement: S1 Table — Due to small cell counts, some data are not reported (indicated by an NA). OUD = opioid use disorder; MOUD = medication for opioid use disorder. (PDF) [file pone.0341739.s001.pdf]

State	Prescribed buprenorphine, extended-release injectable naltrexone, or methadone within 14 days of OUD diagnosis (N)	Diagnosed with an OUD and continuously enrolled in Medicaid for 14 days after diagnosis (N)	Initation metric (%)	Initiated MOUD within 14 days of OUD diagnosis and received >2 services within 30 days of MOUD initiation (N)	Diagnosed with an OUD and continuously enrolled in Medicaid for 44 days (N)	Engagement metric (%)	Received at least 180 days of continuous MOUD therapy (no gaps in MOUD treatment > 7 days) (N)	Diagnosed with an OUD, initiated MOUD, and continuously enrolled in Medicaid for at least 180 days after initiating MOUD (N)	Retention metric (%)
AR	147	959	15.3	35	862	3.6	21	66	31.8
AZ	1408	7252	19.4	976	6589	13.5	101	999	10.1
CA	4337	19701	22	3104	18049	15.8	552	3253	17
CO	1422	5091	27.9	680	4673	13.4	192	908	21.1
CT	1410	4951	28.5	1040	4620	21	398	1007	39.5
DE	520	1841	28.2	403	1721	21.9	112	329	34
HI	52	435	12	14	395	3.2	NA	41	NA
IA	129	1185	10.9	76	1101	6.4	25	96	26
IL	1192	9103	13.1	494	8373	5.4	260	886	29.3
KY	5518	15507	35.6	4197	14402	27.1	1203	3736	32.2
MA	2212	6454	34.3	1537	5948	23.8	569	1551	36.7
MI	1249	8868	14.1	684	8143	7.7	338	901	37.5
MN	561	2875	19.5	365	2580	12.7	125	343	36.4
ND	44	251	17.5	27	227	10.8	NA	34	NA
NH	895	1585	56.5	370	1463	23.3	314	666	47.1
NJ	1653	8585	19.3	961	7892	11.2	250	1112	22.5
NM	1568	3736	42	969	3469	25.9	205	966	21.2
NV	295	2065	14.3	122	1871	5.9	34	185	18.4
NY	2808	17251	16.3	1257	15818	7.3	803	2147	37.4
OH	5203	17352	30	3564	15916	20.5	1040	3562	29.2
OR	898	3431	26.2	599	3143	17.5	189	608	31.1
RI	187	428	43.7	128	388	29.9	33	107	30.8
VT	355	822	43.2	223	760	27.1	78	240	32.5
WA	3814	9966	38.3	2294	9276	23	475	2527	18.8
WV	3038	5441	55.8	2107	5067	38.7	831	1689	49.2
