# Supplementary material for: Variation in the quality of opioid use disorder treatment in the Medicaid population in 2019
Source: PLoS One. 2026 Mar 4;21(3):e0341739. doi: 10.1371/journal.pone.0341739 (PMC12959680; doi:10.1371/journal.pone.0341739)
Supplement: S2 Table — OUD = opioid use disorder; MOUD = medication for opioid use disorder. (PDF) [file pone.0341739.s002.pdf]

Urbanicity classification	Prescribed buprenorphine, extended-release injectable naltrexone, or methadone within 14 days of OUD diagnosis (N)	Diagnosed with an OUD and continuously enrolled in Medicaid for 14 days after diagnosis (N)	Initation metric (%)	Initiated MOUD within 14 days of OUD diagnosis and received >2 services within 30 days of MOUD initiation (N)	Diagnosed with an OUD and continuously enrolled in Medicaid for 44 days (N)	Engagement metric (%)	Received at least 180 days of continuous MOUD therapy (no gaps in MOUD treatment > 7 days) (N)	Diagnosed with an OUD, initiated MOUD, and continuously enrolled in Medicaid for at least 180 days after initiating MOUD (N)	Retention metric (%)
Urban	29731	119360	24.9	18872	109705	15.8	5462	20454	26.7
Suburban	6281	19397	32.4	3983	17902	20.5	1513	4269	35.4
Rural	3892	11157	34.9	2764	10356	24.8	1000	2554	39.2
Missing	1011	5221	19.4	607	4783	11.6	190	682	27.9
